# Longitudinal plasma inflammatory proteome profiling during pregnancy in the Born into Life study

**DOI:** 10.1038/s41598-020-74722-5

**Published:** 2020-10-20

**Authors:** Anna M. Hedman, Cecilia Lundholm, Ellika Andolf, Göran Pershagen, Tove Fall, Catarina Almqvist

**Affiliations:** 1 Department of Medical Epidemiology and Biostatistics, Karolinska Institutet, PO Box 281, 171 77 Stockholm, Sweden; 2 Department of Clinical Sciences, Danderyd Hospital, Stockholm, Sweden; 3 Institute of Environmental Medicine, Karolinska Institutet, Stockholm, Sweden; 4 Department of Medical Sciences, Molecular Epidemiology and Science for Life Laboratory, Uppsala University, Uppsala, Sweden; 5 Pediatric Allergy and Pulmonology Unit, Astrid Lindgren Children’s Hospital, Karolinska University Hospital, Stockholm, Sweden

**Keywords:** Biomarkers, Medical research, Molecular medicine

## Abstract

The maternal immune system is going through considerable changes during pregnancy. However, little is known about the determinants of the inflammatory proteome and its relation to pregnancy stages. Our aim was to investigate the plasma inflammatory proteome before, during and after pregnancy. In addition we wanted to test whether maternal and child outcomes were associated with the proteome. A cohort of 94 healthy women, enrolled in a longitudinal study with assessments at up to five time points around pregnancy, ninety-two inflammatory proteins were analysed in plasma with a multiplex Proximity Extension Assay. First, principal components analysis were applied and thereafter regression modelling while correcting for multiple testing. We found profound shifts in the overall inflammatory proteome associated with pregnancy stage after multiple testing (*p* < .001). Moreover, maternal body mass index (BMI) was associated with inflammatory proteome primarily driven by VEGFA, CCL3 and CSF-1 (*p* < .05). The levels of most inflammatory proteins changed substantially during pregnancy and some of these were related to biological processes such as regulation of immune response. Maternal BMI was significantly associated with higher levels of three inflammation proteins calling for more research in the interplay between pregnancy, inflammation and BMI.

## Introduction

Pregnancy is characterized by substantial changes in the maternal immune system. The allograft paradigm states that the maternal immune system must acquire an anti-inflammatory state during pregnancy in order to prevent rejection of the fetus^1^. Maladaptations during pregnancy underlie pathologies such as preeclampsia, preterm birth and intrauterine growth restriction^2–4^. Early detection of deviations from a healthy pregnancy is of utmost importance since targeted interventions may be applied before clinical expressions become apparent.

Pregnant women exhibit elevated serum levels of certain proinflammatory cytokines compared to those not pregnant^5^. A healthy pregnancy is characterized by mild increase in both serum pro- and anti-inflammatory cytokine levels^6–8^. Inflammation has been proposed as one of the mechanisms involved in labor, both term and preterm^9^ and it has been put forward that early pregnancy and late pregnancy are inflammatory states while mid-pregnancy is an anti-inflammatory process^10^. Proteins are important executors of biological processes, as they include enzymes^11^, are involved in coagulation^12^, provide structural components of tissues^13^ and both prevent and accelerate inflammation^14^.

A few studies have investigated longitudinal changes in proteins during pregnancy in healthy women^15–20^. However, most studies suffer from small sample sizes^15,16,19–25^ in selected study populations^15,17,24,25^. Some studies have included measurements postpartum^20,21,23–25^ in addition to those during pregnancy but no study has included an assessment before conception. Thus, the normal course of change in serum inflammatory proteins before, during and after pregnancy is not fully understood. Analysis of plasma proteins has the potential to detect deviations from normal pregnancy and fetal development.

Maternal factors such as aging could influence the proteome during pregnancy^26^. Moreover, adiposity measured as body mass index (BMI) is generally regarded as contributing to an inflammatory state^27^. In addition, a few studies have shown differences by fetal sex in that women carrying a male fetus display a more pro-inflammatory environment than women carrying a female fetus^28^. In contrast, women carrying females versus males may present greater stimulated cytokine production throughout pregnancy^17^. Furthermore, the maternal plasma proteome has been found to change as a function of the length of gestation^15,21^ and mothers diagnosed with fetuses small for gestational age^29^ and intrauterine growth restriction^30^ have shown altered proteome profile^29,30^. In addition, placental proteins have been found to be associated to onset of labour and mode of delivery^31^. Taken together, both maternal factors, fetal sex and birth outcomes have been associated with certain proteins during pregnancy or delivery and deviations from a healthy pregnancy/delivery might be expressed in inflammation proteins related to these determinants^26,27,29–35^.

Our aim was to investigate the plasma inflammatory proteome before, during and after pregnancy in a cohort of healthy women undergoing pregnancy. Specifically, we aimed to test whether (1) different stages of pregnancy, maternal age, BMI and sex of the fetus is associated with the inflammation proteome (2) the inflammatory proteome can predict the birth outcomes birthweight, gestational age at birth and cesarean section.

## Results

Descriptive characteristics of the sample population are given in Table 1. Mean age at delivery was 32.5 years and mean maternal BMI was 22.9 kg/m^2^. Mean birthweight was 3529 g, mean gestational age was 39.7 weeks and 17 deliveries were performed with cesarean section (18%) while twelve of these were emergency cesarean section (13%).

**Table 1:** Descriptive statistics of women participating in the Born into Life study.

	n (%)	m (SD)
**Age at delivery, y**	94 (100)	32.5 (3.5)
≤ 29	17 (18)	
> 29– ≤ 34	53 (56)	
> 34	24 (26)	
**Maternal BMI**^a^**, kg/m**^**2**^	94 (100)	22.9 (3.4)
< 25	73 (78)	
≥ 25	21 (22)	
**Sample per timepoint**		
Baseline	61 (65)	
w10–14	67 (71)	
w26–28	91 (97)	
Admission	70 (74)	
3 days postpartum	74 (79)	
**Sex of child**		
Boy	56 (60)	
Girl	38 (40)	
**Birthweight, g**	94 (100)	3529 (488)
**Gestational age, w**	94 (100)	39.7 (1.7)
**Caesarian section**	17 (18)	
Emergency	12 (13)	
No emergency	5 (5)	
**Preeclampsia, ICD-10**	1 (1)^b^	
**Education**		
High-school, 10–12 years	8 (9)	
University, > 13 years	77 (82)	
Other	3 (3)	
Missing	6 (6)	

*n* number, *m* mean, *SD* standard deviation, *y* years, *kg/m*^*2*^ kilogram per square-meter, *g* gram, *w* weeks.

^a^Maternal BMI recorded at first antenatal care visit.

^b^Mild to moderate preeclampsia according to ICD-10 = O.14.

**Figure 1:**
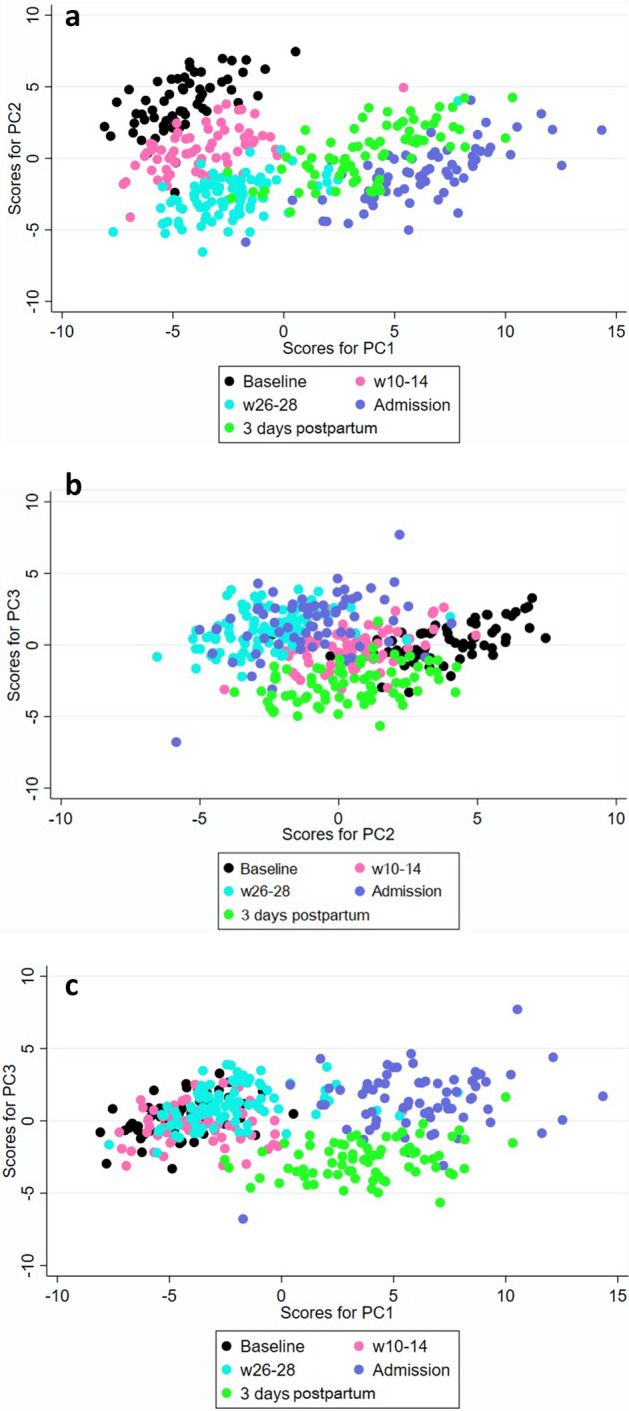
Scatter plots of the first three principal components by time-point. (**a**) PC1 versus PC2, (**b**) PC2 versus PC3, (**c**) PC1 versus PC3. PC = principal component, w = week, admission = admission to delivery ward.

Figure 1a–c illustrate scatter plots of the first three PCs with different colors for each time-point. PCs were based on z-values by raw and logged data for some proteins that were not normally distributed. We went on with 3 PCs since they captured most of the explained variance.

Values for the different time-points are fairly well separated in Fig. 1a which captures most of the variance, indicating that time-point is an important influencing factor for the protein levels.

### Protein change during pregnancy

**Figure 2:**
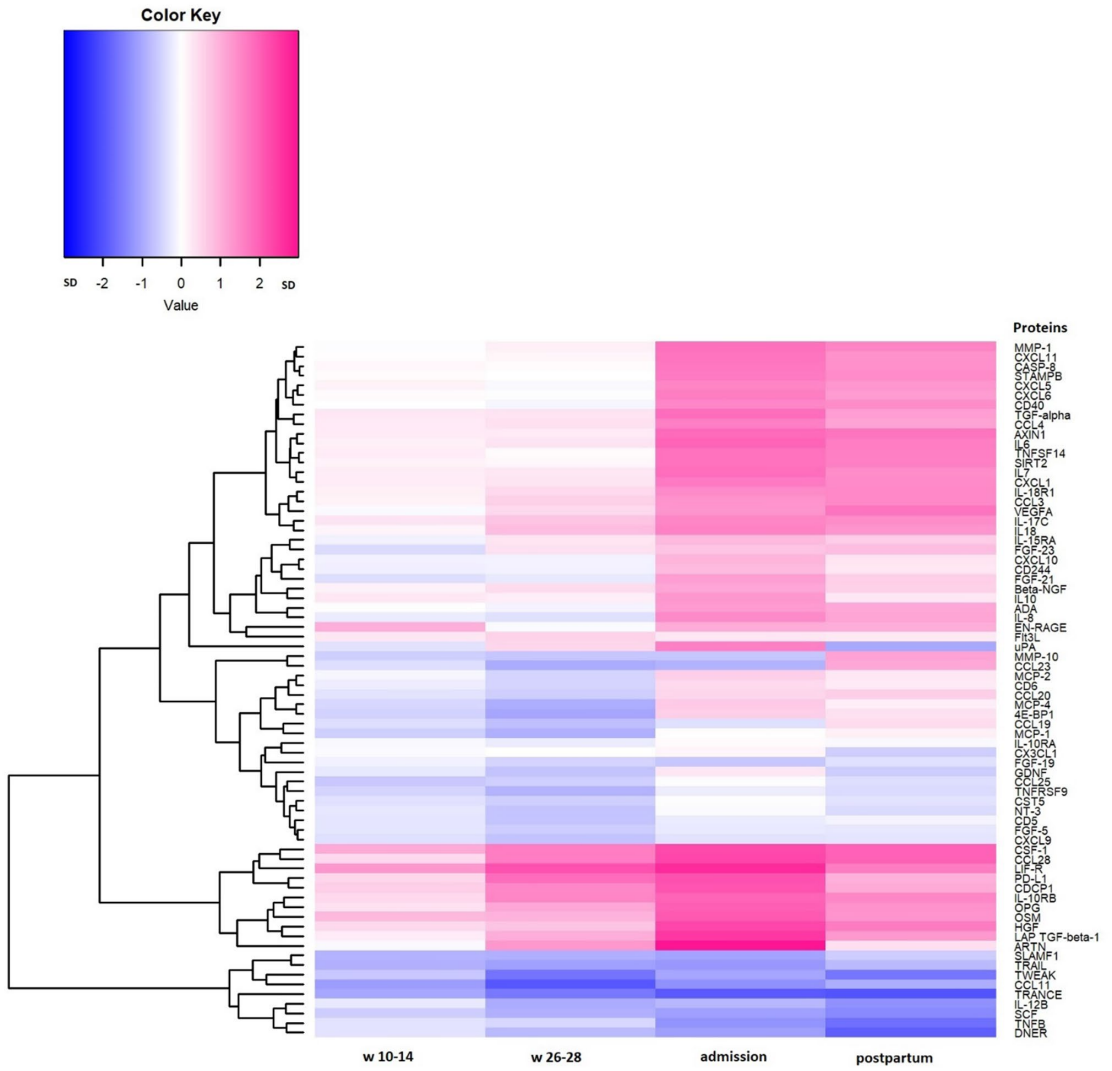
Heatmap of the relative intensity, *β*, between individual protein level and time-point compared to baseline. Color-coding ranges from dark blue = negative *β* to intense rose = positive *β* with white = zero value of *β* in SD (Standard Deviation) units. w = week, 3 days after = 3 days postpartum.

We found differences between the sampling time-points for all three PCs, *p* values < 0.001 (Supplementary Table S1). In the subsequent analyses of individual proteins, we found an association of sampling time with 72 out of 73 individual proteins (Fig. 2 and Table 2).

**Table 2:** Individual protein levels at different time-points compared to baseline expressed as *β*-value (difference in means between each time point and baseline) in SD units.

Protein	w10–14		w26–28		Admission		3 days postpartum		*p* (Wald chi^2^ test)	*p* FDR
	*β*	*p*	*β*	*p*	*β*	*p*	*β*	*p*		
IL-8	− 0.23	0.03	− 0.36	< .01	1.48	< .01	1.15	< .01	< .0001	< .0001
EN-RAGE	1.00	< .01	− 0.05	0.73	1.04	< .01	1.02	< .01	< .0001	< .0001
VEGFA	− 0.04	0.73	0.49	< .01	1.34	< .01	1.76	< .01	< .0001	< .0001
BDNF	− 0.07	0.29	− 0.07	0.26	− 0.03	0.66	− 0.09	0.20	0.6700	0.6700
GDNF	− 0.25	0.04	− 0.70	< .01	0.28	0.02	− 0.59	< .01	< .0001	< .0001
CDCP1	0.62	< .01	1.51	< .01	2.15	< .01	1.06	< .01	< .0001	< .0001
CD244	− 0.19	0.18	− 0.16	0.23	0.84	< .01	0.28	0.04	< .0001	< .0001
IL7	0.23	0.04	0.28	0.01	1.84	< .01	1.44	< .01	< .0001	< .0001
OPG	0.36	< .01	1.10	< .01	2.01	< .01	1.38	< .01	< .0001	< .0001
LAP TGF-beta-1	0.25	< .01	1.03	< .01	2.52	< .01	1.31	< .01	< .0001	< .0001
uPA	− 0.31	< .01	0.51	< .01	1.60	< .01	− 1.00	< .01	< .0001	< .0001
IL6	0.18	0.25	0.32	0.03	1.95	< .01	1.64	< .01	< .0001	< .0001
IL-17C	0.31	0.09	0.74	< .01	1.54	< .01	1.44	< .01	< .0001	< .0001
MCP-1	− 0.54	< .01	− 0.92	< .01	0.01	0.92	0.19	0.11	< .0001	< .0001
CXCL11	0.02	0.88	0.13	0.18	1.76	< .01	1.37	< .01	< .0001	< .0001
AXIN1	0.26	0.03	0.26	0.02	1.89	< .01	1.76	< .01	< .0001	< .0001
TRAIL	− 0.91	< .01	− 1.10	< .01	− 1.19	< .01	− 0.80	< .01	< .0001	< .0001
CXCL9	− 0.37	0.01	− 0.69	< .01	− 0.32	0.03	− 0.29	0.04	0.0003	0.0003
CST5	− 0.34	< .01	− 0.55	< .01	− 0.02	0.84	− 0.31	< .01	< .0001	< .0001
OSM	0.89	< .01	0.96	< .01	2.10	< .01	1.37	< .01	< .0001	< .0001
CXCL1	0.24	0.03	0.34	< .01	1.68	< .01	1.49	< .01	< .0001	< .0001
CCL4	0.26	0.01	0.38	< .01	1.60	< .01	1.15	< .01	< .0001	< .0001
CD6	− 0.21	0.16	− 0.49	< .01	0.45	< .01	0.26	0.08	< .0001	< .0001
SCF	− 0.59	< .01	− 0.92	< .01	− 1.11	< .01	− 1.35	< .01	< .0001	< .0001
IL18	0.12	0.21	0.82	< .01	1.58	< .01	1.35	< .01	< .0001	< .0001
SLAMF1	− 0.87	< .01	− 0.93	< .01	− 1.05	< .01	− 0.58	< .01	< .0001	< .0001
TGF-alpha	0.29	0.01	0.31	0.01	1.83	< .01	1.22	< .01	< .0001	< .0001
MCP-4	− 0.45	< .01	− 0.95	< .01	0.66	< .01	0.22	0.06	< .0001	< .0001
CCL11	− 1.14	< .01	− 1.95	< .01	− 1.28	< .01	− 0.95	< .01	< .0001	< .0001
TNFSF14	0.24	0.02	0.05	0.59	1.77	< .01	1.62	< .01	< .0001	< .0001
FGF-23	− 0.42	< .01	0.36	< .01	0.74	< .01	0.81	< .01	< .0001	< .0001
IL-10RA	− 0.07	0.42	− 0.22	0.01	0.05	0.58	− 0.05	0.53	0.0079	0.0080
FGF-5	− 0.30	< .01	− 0.58	< .01	− 0.24	< .01	− 0.27	< .01	< .0001	< .0001
MMP-1	− 0.02	0.81	0.17	0.03	1.79	< .01	1.54	< .01	< .0001	< .0001
LIF-R	1.30	< .01	2.20	< .01	2.67	< .01	1.64	< .01	< .0001	< .0001
FGF-21	− 0.39	< .01	− 0.24	0.03	1.22	< .01	0.62	< .01	< .0001	< .0001
CCL19	− 0.39	< .01	− 0.74	< .01	− 0.36	< .01	0.43	< .01	< .0001	< .0001
IL-15RA	− 0.17	0.16	0.33	< .01	0.91	< .01	0.64	< .01	< .0001	< .0001
IL-10RB	0.47	< .01	1.55	< .01	1.96	< .01	1.51	< .01	< .0001	< .0001
IL-18R1	0.19	0.03	0.48	< .01	1.47	< .01	1.49	< .01	< .0001	< .0001
PD-L1	0.50	< .01	1.86	< .01	2.20	< .01	0.98	< .01	< .0001	< .0001
Beta-NGF	0.16	0.13	0.42	< .01	1.13	< .01	0.57	< .01	< .0001	< .0001
CXCL5	0.13	0.25	− 0.07	0.54	1.54	< .01	1.32	< .01	< .0001	< .0001
TRANCE	− 1.01	< .01	− 1.55	< .01	− 1.92	< .01	− 1.98	< .01	< .0001	< .0001
HGF	0.46	< .01	0.74	< .01	2.33	< .01	1.66	< .01	< .0001	< .0001
IL-12B	− 0.23	0.01	− 0.95	< .01	− 0.85	< .01	− 1.27	< .01	< .0001	< .0001
ARTN	− 0.06	0.70	1.30	< .01	2.99	< .01	0.40	< .01	< .0001	< .0001
MMP-10	− 0.53	< .01	− 0.66	< .01	− 0.66	< .01	1.15	< .01	< .0001	< .0001
IL10	0.28	0.06	0.23	0.09	1.31	< .01	0.28	0.05	< .0001	< .0001
CCL23	− 0.38	< .01	− 0.97	< .01	− 0.87	< .01	1.09	< .01	< .0001	< .0001
CD5	− 0.29	0.04	− 0.67	< .01	− 0.21	0.14	− 0.13	0.37	< .0001	< .0001
CCL3	0.16	0.17	0.58	< .01	1.35	< .01	1.50	< .01	< .0001	< .0001
Flt3L	0.29	0.03	0.55	< .01	0.27	0.04	0.25	0.05	0.0002	0.0002
CXCL6	0.06	0.60	− 0.03	0.76	1.63	< .01	1.24	< .01	< .0001	< .0001
CXCL10	− 0.16	0.21	− 0.13	0.28	0.94	< .01	0.34	0.01	< .0001	< .0001
4E-BP1	− 0.52	< .01	− 1.04	< .01	0.59	< .01	0.35	0.01	< .0001	< .0001
SIRT2	0.13	0.23	0.11	0.30	1.77	< .01	1.61	< .01	< .0001	< .0001
CCL28	0.48	< .01	1.64	< .01	2.33	< .01	2.00	< .01	< .0001	< .0001
DNER	− 0.32	< .01	− 0.80	< .01	− 1.11	< .01	− 1.86	< .01	< .0001	< .0001
CD40	− 0.01	0.95	− 0.10	0.35	1.52	< .01	1.45	< .01	< .0001	< .0001
FGF-19	− 0.15	0.36	− 0.50	< .01	− 0.66	< .01	− 0.37	0.02	0.0001	0.0001
MCP-2	− 0.09	0.29	− 0.50	< .01	0.59	< .01	0.30	< .01	< .0001	< .0001
CASP-8	0.09	0.42	0.06	0.59	1.71	< .01	1.40	< .01	< .0001	< .0001
CCL25	− 0.65	< .01	− 0.55	< .01	− 0.01	0.89	− 0.37	< .01	< .0001	< .0001
CX3CL1	− 0.04	0.69	0.01	0.94	0.12	0.28	− 0.56	< .01	< .0001	< .0001
TNFRSF9	− 0.50	< .01	− 0.87	< .01	− 0.21	0.12	− 0.41	< .01	< .0001	< .0001
NT-3	− 0.29	0.05	− 0.67	< .01	− 0.04	0.78	− 0.42	< .01	< .0001	< .0001
TWEAK	− 0.64	< .01	− 1.62	< .01	− 1.03	< .01	− 1.60	< .01	< .0001	< .0001
CCL20	− 0.32	0.02	− 0.56	< .01	0.51	< .01	0.59	< .01	< .0001	< .0001
STAMPB	0.04	0.70	< .01	0.97	1.68	< .01	1.46	< .01	< .0001	< .0001
ADA	0.01	0.91	− 0.13	0.22	1.25	< .01	1.09	< .01	< .0001	< .0001
TNFB	− 0.32	< .01	− 0.45	< .01	− 1.24	< .01	− 1.65	< .01	< .0001	< .0001
CSF-1	1.06	< .01	1.65	< .01	2.30	< .01	1.98	< .01	< .0001	< .0001

*FDR* false discovery rate, *β* beta coefficient, *SD* standard deviation, *w* week.

Higher color intensity indicates larger mean difference (in standard deviation units) compared to baseline (*β*). Pink color stands for higher mean protein-level compared to baseline and blue color stands for lower levels. The side dendrogram shows which proteins behave most similarly. The uppermost part of the heatmap and the second lowest part show proteins with higher (pink) protein-level at time-points during/after pregnancy compared to baseline, whereas the lowest part of the dendrogram shows the proteins with lower (blue) protein-levels at those time-points compared to baseline. The proteins in the middle of the dendrogram have a mixture of higher and lower protein levels for different time-points compared to baseline. Throughout the dendrogram there are proteins (white) with one or more time-points with no difference in mean protein-level compared to baseline.

Several proteins in the TNF-family were included in our study (TRAIL, TRANCE, TNF-beta, TWEAK, TNFSF14, TNFRSF9, OPG and CD40). The majority of the measured TNF-related proteins (TRAIL, TRANCE, TNF-beta and TWEAK and to a lesser extent TNFRSF9) showed very similar patterns over the sampling points in our study and were strongly down-regulated during and after pregnancy. The pro-inflammatory cytokine TNFSF14 and OPG was in contrast strongly upregulated.

Another important protein family which is well presented in our study are the interleukins, ILs (-7, -10, -10RA, -10RB, -12B, -15RA, -17C, -18, -18R1). Most of our proteins from the IL-family that survived multiple correction were up-regulated during and after pregnancy (IL-7/-10/-10RB/-17C/-18/-18R1) while the subunit of interleukin 12 (IL-12B) was down-regulated at all time-points. IL-15RA decreased in the first sampling point but then increased in the other time-points compared to baseline and IL-10RA was slightly down-regulated except for the time around admission.

Many proteins belonging to the chemokine-family were investigated in our study (MCP-1/-2/-4, CCL-3/-4/-11/-19/-20/-23/-25/-28, CXCL-1/5/-6/-8/-9/-10/-11). We found a U-shaped pattern between the time-points of IL-8. MCP-1 increased from during pregnancy to postpartum and CCL-25 and CCL-28 increased from early to late pregnancy. CXCL-6 had no change in early pregnancy and after that an increase at the admission time-point and after.

Other important cytokines included in our study are CSF-1, LIF-R and IL6. Here we found increased levels of CSF-1 during and after pregnancy compared to pre-pregnancy. LIF-R increased during and after pregnancy (Supplementary Table S2) and IL-6 continuously increased compared to baseline at w 26–28 in pregnancy and 3 days postpartum.

### Determinants of protein levels

We found evidence of an association between maternal BMI and level of first PC (PC1) adjusted for maternal age at delivery and time-point (*p* = 0.03), Supplementary Table S2. No significant associations were seen between maternal age at delivery and the PCs, Supplementary Table S2.

BMI was positively associated with Vascular endothelial growth factor A, VEGFA (*β* = 0.05, *p* < 0.001), Chemokine ligand 3, CCL3 (*β* = 0.06, *p* < 0.001) and Colony stimulating factor 1, CSF-1 (*β* = 0.06, *p* < 0.001) in models adjusted for maternal age at delivery and time-point after FDR correction. The regression coefficients and corresponding p-values for all individual proteins and maternal BMI are found in Supplementary Table S3. No significant interaction between maternal BMI and time-point was seen.

There were no significant associations between maternal BMI, age at delivery or fetal sex and change of proteins from baseline, Supplementary Table S4.

### Proteomic prediction of birth outcomes

There were no significant associations between protein level at baseline (Supplementary Table S5a), protein-level w 10–14 (Supplementary Table S5b) or protein-level at w 26–28 (Supplementary Table S5c) and birth outcomes (i.e., birthweight, gestational age, cesarean section/-emergency), adjusted for maternal BMI. In addition, no significant associations were found between protein level at w 26–28 and birth outcomes controlling for baseline protein-level, maternal BMI (Supplementary Table S5d) and protein-level w 10–14 (Supplementary Table S5e).

No association was seen between protein change (between baseline and w 26–28) and any of the birth outcomes (i.e., birthweight, gestational age, cesarean section/-emergency), adjusted for maternal BMI (Supplementary Table S6).

### Sensitivity analysis

No difference in results were found after excluding one woman with pre-eclampsia (data not shown).

## Discussion

In this study we investigated the maternal plasma inflammation proteome levels before, during and after pregnancy in healthy women and association with maternal factors and birth outcomes.

We found significant changes in the proteome during pregnancy compared to baseline in the majority of the proteins. The results confirm the immense change in immune function and inflammation status during the pre-, peri- and postnatal period. Furthermore, maternal BMI was significantly associated with increased protein levels of VEGFA, CCL3 and CSF-1. We did not find any association between the proteome and birth outcomes. To our awareness, this is the first study with assessment before conception that investigates the plasma proteome longitudinally during and after pregnancy.

### Protein change during pregnancy

The TNF superfamily of proteins is expressed predominantly by immune cells regulating diverse cell functions, including immune response and inflammation, but also proliferation, differentiation, apoptosis and embryogenesis. A recent Swedish study investigated the inflammatory proteome with the same technology in 22 pregnant women followed longitudinally at two time-points from late pregnancy to week 8 postpartum and found lower levels of TWEAK, TRANCE and TRAIL and higher levels TNFSF14 and OPG during pregnancy in accordance with our findings^20^. In contrast, in their study TNF-beta was higher in pregnancy and TNFRSF9 unchanged but CD40 appeared similar to their results if only comparing our time-points from admission to postpartum.

The interleukin proteins are highly related to suppression and activation of the immune system and cell division. In line with Bränn et al.^20^ we found a decrease between admission and postpartum in IL-7/-10RB/-15RA and IL-17C. In contrast, we found a slight increase between admission and postpartum in IL-18R1 and a decrease between the same time-points in IL-12B while Bränn et al.^20^ found a decrease in IL-18R1 and an increase in IL-12B. However, another study investigating IL-12B longitudinally found elevated levels in cord-blood but then a decrease to postpartum and thereafter a slight increase a few months after delivery^36^ supporting our data from admission to postpartum. Wang et al.^37^ found increased level of IL-18 in pregnant women compared to non-pregnant women confirming our results of increased levels of IL-18 throughout pregnancy and postpartum compared to baseline. A few other longitudinal studies are in accordance with our result of a decrease in IL-10 between late pregnancy and postpartum^16,20,36^. In addition, another study found stability in IL-10 postpartum measured at multiple time-points^8^.

Chemokines control the homeostasis of the immune cells and represent a pro-inflammatory mediator. One of the most studied chemokines longitudinally in pregnancy is the IL-8 protein (also named CXCL-8) where several studies found no change during pregnancy^16,17,25^ or from late pregnancy to postpartum^8,25^. In line with our U-shaped pattern between the time-points of IL-8 is Christian et al.^23^ who found decreased level of IL-8 during pregnancy but increased level postpartum. Also in accordance with our results of MCP-1 are findings of increase from during pregnancy to postpartum^8,22^. Romero et al.^15^ investigated the maternal plasma proteome changes and found that CCL-25 and CCL-28 were increasing from early to late pregnancy which is in line with our results on the same chemokines. In contrast, Romero et al.^15^ found that CXCL-6 decreased from early to late pregnancy while we found no change in early pregnancy and after that an increase to the admission time-point.

CSF-1 influences several processes involved in immunology, fertility and pregnancy. Our results are in accordance with another study which found two-fold enhanced levels of CSF-1 during pregnancy where the authors strongly argued about an important role in gestation^38^. LIF-R, which maintain stem cell pluripotency, showed the greatest changes in the study by Bränn et al.^20^ with higher LIF-R in pregnancy compared to postpartum^20^. This was confirmed in our study where we found that LIF-R increased during pregnancy (Supplementary Table S2). IL-6, which is a pro-inflammatory cytokine, has also been greatly investigated during pregnancy where most studies found increasing values across pregnancy^19,23,25^ and mixed results from late pregnancy to postpartum^23,25^. In our study IL-6 significantly continuously increased compared to baseline at w 26–28 in pregnancy and 3 days postpartum. Thus, results of studies indicate that some increases in IL-6 during pregnancy seem to occur but results regarding postpartum are difficult to compare since studies investigate different weeks of gestation and different length postpartum^23,25^ or compare the change from pre-pregnancy.

### BMI and proteins

We found that maternal BMI was associated with higher levels of VEGFA, CCL3 and CSF-1 independently of pregnancy stage. Previous research has mainly found associations between obesity and elevated levels in IL-6, IL-8 and Tumor Necrosis Factor alpha^39–42^. Levels of VEGFA and CSF-1 measured in cerebrospinal fluid have been found to be positively associated with BMI^43^ and the VEGFA gene is found to be mediating the connection between obesity and breast cancer^44^. CSF-1 regulates the production, function and differentiation of macrophages^45^ and macrophages have been demonstrated to accompany obesity in adipose tissue^46^. Our result of an association between maternal BMI and the levels of CSF-1 are in line with these findings. This also corroborates the findings from another study which found that level of CSF-1 correlated with the number of omental macrophages which in turn were associated with waist circumference^47^. Moreover, serum protein level of CCL-3 has been found to correlate with visceral white adipose tissue in healthy obese individuals undergoing bariatric surgery^48^. Thus, results suggest a low-grade inflammatory state in VEGFA, CSF-1 and CCL3 which is related to obesity and BMI. Furthermore, obesity is a primary promoter of inflammation and obesity in pregnancy is associated with gestational hypertension and gestational diabetes with inflammation as a probable mechanism^49^. Obesity and pregnancy independently contribute to a state of chronic inflammation and it has been suggested that the combined inflammatory response can be particularly harmful for both the mother and the fetus^39,40^. In light of this, more research is needed to elucidate the mechanism(s) behind altered inflammation in overweight women and its potential interplay with pregnancy.

Limitations of the study include relative measurement of the protein level rather than absolute concentrations. In addition, women included had a relatively low pre-pregnancy BMI, few adverse pregnancy outcomes and high education, which may limit the generalizability. Furthermore, we had a relatively small sample and hence limited statistical power. However, we utilized robust statistical modelling with control for multiple testing and applied sensitivity-analyses excluding the woman diagnosed with preeclampsia yielding the same results giving us reliance in our findings. We had a rather limited number of proteins compared to some^15,21^ but not all^20^ proteomic studies. In regards to number of longitudinal samples collected during pregnancy, some studies have reported more^8,15,18,21^ and others less^16,20,22,50^ time-points compared to our study. But, our study had a two to threefold number of included participants compared to recent longitudinal studies investigating proteins in pregnant women^15,20,21^. Although we included multiple time-points, ideally an assessment one year postpartum would be optimal to follow whether protein level go back to baseline (i.e., before conception). Strengths of the study include novel technique with a high-dimensional Proximity Extension Assay-method which is more sensitive than 2-dimensional gel or mass spectrometry and can detect analytes in very small quantities^51^. Another strength is assessments before as well as after pregnancy in addition to the three time-points during pregnancy. To our knowledge, this is the first study that includes a baseline measurement before conception, which is of importance as a reference in non-pregnant women.

### Conclusions

Here we add another piece of the puzzle on how inflammatory proteins vary over time in pregnant women mainly by including a baseline measure before conception. Thus, changes were substantial compared to baseline in this cohort of healthy pregnant women making this study unique. Maternal BMI was significantly associated with higher levels of three inflammation proteins, calling for more research in the interplay between pregnancy, inflammation and BMI.

## Methods

### Study design and study sample

This study was based on the prospective longitudinal cohort study—Born into Life—following women from Stockholm, Sweden, before, during and after pregnancy and their children pre-, peri-, and postnatally^52^. Born into Life was recruited from the larger LifeGene study which has been described in detail elsewhere^53^. In short, LifeGene included index persons between 18–45 years who were also encouraged to invite their family members. At enrollment (baseline), biosamples were taken and a web-based questionnaire with multifaceted questions regarding health, lifestyle and diseases was administered. Women who became pregnant in LifeGene were invited to Born into Life and we included all those who answered a web-based questionnaires at gestational week 10–14 and 26–28 regarding health, diseases, lifestyle and pregnancy. Ethical approval has been obtained by the Regional Ethics Review Board in Stockholm, Sweden and all study participants have given informed consent. All research was performed in accordance with relevant guidelines and regulations.

Originally, 107 women were included in Born into Life. The present study required that each woman had at least one serum measurement during pregnancy. In total, 94 women were included.

### Data sources and variables

Information on age, sex, gestational age, birthweight, cesarean section and emergency cesarean section was retrieved from the medical records. Age was recorded at the time of delivery and categorized into ≤ 29 years, 30–34 years and > 34 years. Gestational age was measured in weeks and birthweight in grams. BMI was based on weight and height measurements collected from the first antenatal visit and grouped into < 25 kg/m^2^ and ≥ 25 kg/m^2^. As a proxy for socioeconomic status, self-reported education in years was used with the following categorization: 10–12 years, > 12 years, and “other”. Venous blood samples (EDTA tubes) were taken at five time-points: baseline, during pregnancy week 10–14 and week 26–28, at admission to hospital and 3 days after delivery.

### Proteomic profiling and quality control

Plasma was separated from blood samples, aliquoted and stored at − 80 °C until analysis. Plasma samples were analysed for relative levels of 92 inflammation-linked proteins using the Olink multiplex arrays (INFII panel, Proseek multiplex arrays; Olink Bioscience, Uppsala, Sweden) according to the manufacturer’s instructions^51,54^. In brief, a standard 96-well microplate format is used by the assay, including 90 samples and six external quality control standards. Ninety-two oligonucleotide-labelled pairs of antibodies were mixed with each sample. When high-specificity antibodies bind the target protein, the attached oligonucleotides form a unique DNA reporter sequence which is subsequently quantified and amplified with standard PCR. Samples were randomly distributed and analysed on seven plates. PCR values above the fluorescence detection limit were log2-transformed and corrected for technical variations based on negative and inter-plate controls. With the negative control samples (buffer with antigen) the lower limits of detection (LOD) were determined. For the quality control, nine samples had no measurements and 19 proteins (MCP-3, IL-17A, IL-20RA, IL-2RB, IL-1 alpha, IL2, TSLP, IL-22 RA1, IL-24, IL-13, TNF, IL-20, IL-33, INF-gamma, IL4, LIF, NRTN, ST1A1, IL-5) had > 15% of measurements below LOD, and were removed. The final data set included 73 proteins. Principal components (PCs) were calculated based on levels of the 73 proteins that passed the quality control, with values below LOD imputed to LOD/2.

Linear regression analysis of the PCs indicated that the protein levels were associated with storage time but not with plate. Consequently, all proteins were normalized for storage time, before re-calculating the PCs. Scatterplots of the first three PCs (PC1-PC3) with different markers for each time-point was plotted to display differences over time in the PCs.

### Statistics

To test whether (1) the potential determinants stage of pregnancy, maternal age, BMI and sex of the fetus are associated with the inflammatory proteome (2) the inflammatory proteome can predict the birth outcomes birthweight, gestational age at birth and cesarean section, we applied the following approach:

1. Each of the potential determinants (stage of pregnancy, maternal age, BMI and sex of the fetus) were tested as independent variables for association with the first three principal components using linear regression modelling with one model for each PC using a threshold of *p* < 0.05 to denote significance.
2. We tested the association of the first three principal components as predictors with each of the birth outcomes (birthweight, gestational age at birth and cesarean section/-emergency) using regression modelling in a within-subjects design and a threshold of *p* < 0.05 to denote significance. We used linear regression for birthweight and gestational age and logistic regression for cesarean section/-emergency adjusted for maternal BMI and a threshold of *p* < 0.05 to denote significance.
3. For those potential determinants found associated with the overall proteome in Step 1, we applied separate regression models for each protein and determinant (independent variable), correcting for multiple testing applying 5% false discovery rate procedure (FDR)^55^. FDR can be seen as the expected proportion of false discoveries, i.e., incorrectly rejected null hypotheses, among all discoveries. We used tobit model versions of ordinary regression or mixed model regression. The tobit model includes values below a LOD without the need of imputation^56^. For association between time-points (stages of pregnancy, baseline as reference) and protein we used the tobit mixed model, with significance based on the omnibus Wald chi2-test. The regression coefficients for each individual protein passing the FDR were presented in a heatmap. For associations between maternal age at delivery or BMI at the first antenatal care visit and protein levels during/after pregnancy we used tobit models with a sandwich estimator adjusting standard errors for time-point. The analyses of maternal BMI were additionally adjusted for maternal age, as a potential confounder. If maternal BMI was found to be significantly associated with protein level, interaction between maternal BMI and time-point were further explored. The association between maternal BMI or age and change in proteins from baseline were analysed in the same way but with the addition of adjustment for baseline value in order to assess change from baseline rather than level. We used the same model for associations between sex of the fetus and change in proteins from baseline.

We identified one woman with mild to moderate preeclampsia, according to International Classification of Diseases (ICD)-codes, version 10=O.14, from the antenatal medical charts. Therefore, as sensitivity analyses we excluded this woman and ran all our analyses again.

All statistical analyses were carried out in STATA, version 15.1.

## Supplementary information

Supplementary Information.

## Data Availability

The datasets generated during and/or analysed during the current study are available from the corresponding author on reasonable request.
