# Supplementary material for: Longitudinal plasma inflammatory proteome profiling during pregnancy in the Born into Life study
Source: Sci Rep. 2020 Oct 20;10:17819. doi: 10.1038/s41598-020-74722-5 (PMC7575597; doi:10.1038/s41598-020-74722-5)
Supplement: Supplementary file 1 — Supplementary Information. [file 41598_2020_74722_MOESM1_ESM.docx]

**Supplementary Information**

**Longitudinal plasma inflammatory proteome profiling during pregnancy in the Born into Life study**

Anna M. HEDMAN, PhD^1^, Cecilia LUNDHOLM, MSc^1^, Ellika ANDOLF, MD, PhD^2^, Göran PERSHAGEN, MD, PhD^3^, Tove FALL, VMD, PhD^4^, Catarina ALMQVIST, MD, PhD^1,5^

**Affiliations:**

^1^ Department of Medical Epidemiology and Biostatistics, Karolinska Institutet, Stockholm, Sweden.

^2^ Department of Clinical Sciences, Danderyd Hospital, Stockholm, Sweden

^3^ Institute of Environmental Medicine, Karolinska Institutet, Stockholm, Sweden

^4^ Department of Medical Sciences, Molecular Epidemiology and Science for Life Laboratory, Uppsala University, Uppsala, Sweden

^5^ Pediatric Allergy and Pulmonology Unit, Astrid Lindgren Children’s Hospital, Karolinska University Hospital, Stockholm, Sweden

**Table S1. Test of differences in PC 1-3 of the protein-levels between time-points.**

| **Outcome** | **Wald chi^2^ value** | **p** |
| --- | --- | --- |
| **PC 1** | 1205.15 | <.0001 |
| **PC 2** | 561.17 | <.0001 |
| **PC 3** | 489.87 | <.0001 |

PC=Principal Component

**Table S2. Associations between maternal BMI and age and protein level (PC1-PC3**

|  | **BMI^1^** | | **Age^2^** | |
| --- | --- | --- | --- | --- |
| **Outcome** | ***β*** | **p** | ***β*** | **p** |
| **PC 1** | 0.10 | 0.03* | 0.03 | 0.42 |
| **PC 2** | 0.04 | 0.14 | 0.04 | 0.22 |
| **PC 3** | 0.01 | 0.57 | -0.03 | 0.25 |

PC=Principal Component, *β*=Beta coefficient, BMI=Body Mass Index at first antenatal care visit, Age=Age at delivery, *=Significant.

^1^ adjusted for maternal age and time-point

^2^ adjusted for time-point

**Table S3. Associations between maternal BMI and individual protein level**

|  | **BMI^1^** |  |  |
| --- | --- | --- | --- |
| **Protein** | ***β*** | **p** | **p FDR** |
| IL-8 | 0.01 | 0.2704 | 0.5123 |
| EN-RAGE | 0.01 | 0.4007 | 0.6662 |
| VEGFA* | 0.05 | 0.0001 | 0.0054* |
| BDNF | 0.08 | 0.0613 | 0.3054 |
| GDNF | 0.00 | 0.9200 | 0.9327 |
| CDCP1 | 0.02 | 0.2737 | 0.5123 |
| CD244 | 0.02 | 0.4289 | 0.6662 |
| IL7 | 0.01 | 0.6083 | 0.7656 |
| OPG | 0.01 | 0.7233 | 0.8277 |
| LAP TGF-beta-1 | -0.02 | 0.0624 | 0.3054 |
| uPA | -0.02 | 0.1960 | 0.4616 |
| IL6 | 0.03 | 0.0047 | 0.0679 |
| IL-17C | 0.00 | 0.8475 | 0.8964 |
| MCP-1 | 0.03 | 0.1907 | 0.4616 |
| CXCL11 | 0.00 | 0.8949 | 0.9202 |
| AXIN1 | 0.00 | 0.7179 | 0.8277 |
| TRAIL | 0.03 | 0.1796 | 0.4616 |
| CXCL9 | 0.04 | 0.0367 | 0.2432 |
| CST5 | -0.01 | 0.5850 | 0.7626 |
| OSM | 0.00 | 0.8595 | 0.8964 |
| CXCL1 | 0.01 | 0.6947 | 0.8277 |
| CCL4 | 0.04 | 0.0293 | 0.2375 |
| CD6 | 0.02 | 0.3817 | 0.6634 |
| SCF | -0.04 | 0.1226 | 0.3750 |
| IL18 | 0.02 | 0.2468 | 0.5123 |
| SLAMF1 | 0.01 | 0.5769 | 0.7626 |
| TGF-alpha | 0.01 | 0.4223 | 0.6662 |
| MCP-4 | 0.01 | 0.7256 | 0.8277 |
| CCL11 | -0.03 | 0.0858 | 0.3298 |
| TNFSF14 | 0.01 | 0.4785 | 0.6809 |
| FGF-23 | 0.00 | 0.8025 | 0.8768 |
| IL-10RA | -0.03 | 0.2302 | 0.5082 |
| FGF-5 | -0.01 | 0.6249 | 0.7731 |
| MMP-1 | 0.01 | 0.4799 | 0.6809 |
| LIF-R | -0.02 | 0.0818 | 0.3298 |
| FGF-21 | 0.03 | 0.1233 | 0.3750 |
| CCL19 | 0.05 | 0.0185 | 0.1928 |
| IL-15RA | 0.04 | 0.1071 | 0.3723 |
| IL-10RB | 0.02 | 0.2232 | 0.5082 |
| IL-18R1 | 0.04 | 0.0287 | 0.2375 |
| PD-L1 | 0.00 | 0.9715 | 0.9715 |
| Beta-NGF | 0.01 | 0.5968 | 0.7644 |
| CXCL5 | 0.02 | 0.2737 | 0.5123 |
| TRANCE | 0.03 | 0.0788 | 0.3298 |
| HGF | 0.01 | 0.4657 | 0.6809 |
| IL-12B | 0.02 | 0.4156 | 0.6662 |
| ARTN | -0.04 | 0.0348 | 0.2432 |
| MMP-10 | -0.01 | 0.5157 | 0.7103 |
| IL10 | 0.02 | 0.1932 | 0.4616 |
| CCL23 | 0.02 | 0.1852 | 0.4616 |
| CD5 | 0.02 | 0.3435 | 0.6269 |
| CCL3* | 0.06 | 0.0009 | 0.0221* |
| Flt3L | 0.06 | 0.0129 | 0.1571 |
| CXCL6 | 0.00 | 0.8525 | 0.8964 |
| CXCL10 | 0.02 | 0.2367 | 0.5082 |
| 4E-BP1 | -0.02 | 0.1434 | 0.4026 |
| SIRT2 | 0.01 | 0.4384 | 0.6667 |
| CCL28 | -0.02 | 0.0990 | 0.3613 |
| DNER | -0.03 | 0.0738 | 0.3298 |
| CD40 | 0.02 | 0.0628 | 0.3054 |
| FGF-19 | -0.02 | 0.2720 | 0.5123 |
| MCP-2 | -0.02 | 0.5422 | 0.7329 |
| CASP-8 | 0.01 | 0.3572 | 0.6360 |
| CCL25 | -0.01 | 0.6963 | 0.8277 |
| CX3CL1 | -0.01 | 0.8048 | 0.8768 |
| TNFRSF9 | 0.04 | 0.0404 | 0.2456 |
| NT-3 | -0.01 | 0.4850 | 0.6809 |
| TWEAK | -0.03 | 0.1229 | 0.3750 |
| CCL20 | 0.05 | 0.0038 | 0.0679 |
| STAMPB | 0.01 | 0.4259 | 0.6662 |
| ADA | 0.01 | 0.7866 | 0.8768 |
| TNFB | 0.14 | 0.1403 | 0.4026 |
| CSF-1* | 0.06 | <.0001 | 0.0002* |
|  |  |  |  |

*β*=Beta coefficient, BMI=Body Mass Index at first antenatal care visit, FDR=False Discovery Rate correction *=significant after FDR

**^1^** adjusted for maternal age at delivery and time-point (omitted in the Table).

**Table S4. Associations between maternal BMI maternal age sex of fetus and protein change from baseline.**

|  | **BMI^1,2^** | | **Age^1^** | | **Sex^1^** | |
| --- | --- | --- | --- | --- | --- | --- |
| **Outcome** | ***β*** | **p** | ***β*** | **p** | ***β*** | **p** |
| **PC 1** | 0.09 | 0.29 | -0.02 | 0.70 | 0.64 | 0.15 |
| **PC 2** | 0.00 | 0.98 | 0.04 | 0.30 | 0.49 | 0.13 |
| **PC 3** | -0.01 | 0.71 | -0.01 | 0.69 | 0.36 | 0.20 |

*β*=Beta coefficient, BMI=Body Mass Index at first antenatal care visit, Age=Maternal age at delivery, Sex=Sex of the fetus where male fetus served as reference, PC=Principal Component.

**^1^** all models was adjusted for baseline protein level and time-point except for baseline (omitted in the Table)

**^2^** BMI model was additionally adjusted for Age

**Table S5a. Associations between protein level at baseline and birth outcomes**

|  | **baseline PC1^1^** | | **baseline PC2^1^** | | **baseline PC3^1^** | | **baseline PC1^1^** | | **baseline PC2^1^** | | **baseline PC3^1^** | |
| --- | --- | --- | --- | --- | --- | --- | --- | --- | --- | --- | --- | --- |
| **Outcome** | ***β*** | **p** | ***β*** | **p** | ***β*** | **p** | **OR** | **p** | **OR** | **p** | **OR** | **p** |
| **BW** | -53.33 | 0.26 | -.36 | 0.99 | 34.06 | 0.56 |  |  |  |  |  |  |
| **GA** | -1.80 | 0.13 | -.40 | 0.73 | -.02 | 0.99 |  |  |  |  |  |  |
| **CS** |  |  |  |  |  |  | 1.00 | 0.99 | 0.96 | 0.87 | 1.71 | 0.11 |
| **Emergency CS** |  |  |  |  |  |  | 1.32 | 0.37 | 0.91 | 0.74 | 1.75 | 0.18 |

*β*=Beta-coefficient, OR=Odds Ratio, BW=Birthweight, GA=Gestational age, CS=cesarean sectio, PC=Principal Component. Categorical values of CS and emergency CS where a case of CS and emergency CS respectively are the references.

**^1^** model was adjusted for maternal BMI and baseline protein level, PC1-PC3 (omitted in the Table).

**Table S5b. Associations between protein level and birth outcomes at w 10-14**

|  | **w10-14 PC1^1^** | | **w10-14 PC2^1^** | | **w10-14 PC3^1^** | | **w10-14 PC1^1^** | | **w10-14 PC2^1^** | | **w10-14 PC3^1^** | |
| --- | --- | --- | --- | --- | --- | --- | --- | --- | --- | --- | --- | --- |
| **Outcome** | ***β*** | **p** | ***β*** | **p** | ***β*** | **p** | **OR** | **p** | **OR** | **p** | **OR** | **p** |
| **BW** | -13.21 | 0.37 | 45.28 | 0.45 | -50.76 | 0.39 |  |  |  |  |  |  |
| **GA** | 0.07 | 0.94 | -0.52 | 0.72 | -1.33 | 0.35 |  |  |  |  |  |  |
| **CS** |  |  |  |  |  |  | 1.32 | 0.14 | 0.61 | 0.10 | 0.96 | 0.90 |
| **Emergency CS** |  |  |  |  |  |  | 1.41 | 0.12 | 0.53 | 0.08 | 0.97 | 0.93 |

*β*=Beta-coefficient, OR=Odds Ratio, BW=Birthweight, GA=Gestational age, CS=cesarean sectio, PC=Principal Component. Categorical values of CS and emergency CS where a case of CS and emergency CS respectively are the references.

**^1^** model was adjusted for maternal BMI and protein level at w10-14, PC1-PC3 (omitted in the Table).

**Table S5c. Associations between protein level at w 26-28 and birth outcomes**

|  | **w26-28 PC1^1^** | | **w26-28 PC2^1^** | | **w26-28 PC3^1^** | | **w26-28 PC1^1^** | | **w26-28 PC2^1^** | | **w26-28 PC3^1^** | |
| --- | --- | --- | --- | --- | --- | --- | --- | --- | --- | --- | --- | --- |
| **Outcome** | ***β*** | **p** | ***β*** | **p** | ***β*** | **p** | **OR** | **p** | **OR** | **p** | **OR** | **p** |
| **BW** | 31.25 | 0.28 | -32.96 | 0.44 | -3.03 | 0.94 |  |  |  |  |  |  |
| **GA** | 0.07 | 0.92 | -0.84 | 0.43 | -0.90 | 0.40 |  |  |  |  |  |  |
| **CS** |  |  |  |  |  |  | 1.14 | 0.36 | 0.77 | 0.24 | 0.93 | 0.75 |
| **Emergency CS** |  |  |  |  |  |  | 1.13 | 0.49 | 0.72 | 0.22 | 0.82 | 0.49 |

*β*=Beta-coefficient, OR=Odds Ratio, BW=Birthweight, GA=Gestational age, CS=cesarean sectio, PC=Principal Component. Categorical values of CS and emergency CS where a case of CS and emergency CS respectively are the references.

**^1^** model was adjusted for maternal BMI and protein level at w26-28, PC1-PC3 (omitted in the Table).

**Table S5d. Associations between protein level at w 26-28 and birth outcomes**

|  | **w26-28 PC1^1^** | | **w26-28 PC2^1^** | | **w26-28 PC3^1^** | | **w26-28 PC1^1^** | | **w26-28 PC2^1^** | | **w26-28 PC3^1^** | |
| --- | --- | --- | --- | --- | --- | --- | --- | --- | --- | --- | --- | --- |
| **Outcome** | ***β*** | **p** | ***β*** | **p** | ***β*** | **p** | **OR** | **p** | **OR** | **p** | **OR** | **p** |
| **BW** | 34.65 | 0.33 | -53.11 | 0.36 | 15.03 | 0.80 |  |  |  |  |  |  |
| **GA** | 0.14 | 0.88 | -1.55 | 0.29 | -0.93 | 0.54 |  |  |  |  |  |  |
| **CS** |  |  |  |  |  |  | 1.18 | 0.34 | 0.79 | 0.38 | 1.02 | 0.93 |
| **Emergency CS** |  |  |  |  |  |  | 1.18 | 0.46 | 0.80 | 0.49 | 0.84 | 0.61 |

*β*=Beta-coefficient, OR=Odds Ratio, BW=Birthweight, GA=Gestational age, CS=cesarean sectio, PC=Principal Component. Categorical values of CS and emergency CS where a case of CS and emergency CS respectively are the references.

**^1^** model was adjusted for maternal BMI and baseline protein level, PC1-PC3 (omitted in the Table).

**Table S5e. Associations between protein level at w 26-28 and birth outcomes**

|  | **w26-28 PC1^1^** | | **w26-28 PC2^1^** | | **w26-28 PC3^1^** | | **w26-28 PC1^1^** | | **w26-28 PC2^1^** | | **w26-28 PC3^1^** | |
| --- | --- | --- | --- | --- | --- | --- | --- | --- | --- | --- | --- | --- |
| **Outcome** | ***β*** | **p** | ***β*** | **p** | ***β*** | **p** | **OR** | **p** | **OR** | **p** | **OR** | **p** |
| **BW** | 11.58 | 0.81 | -6.19 | 0.94 | 42.66 | 0.66 |  |  |  |  |  |  |
| **GA** | -0.28 | 0.81 | -0.33 | 0.87 | -1.46 | 0.52 |  |  |  |  |  |  |
| **CS** |  |  |  |  |  |  | 1.30 | 0.30 | 0.74 | 0.41 | 1.35 | 0.43 |
| **Emergency CS** |  |  |  |  |  |  | 1.11 | 0.82 | 1.24 | 0.76 | 0.80 | 0.70 |

*β*=Beta-coefficient, OR=Odds Ratio, BW=Birthweight, GA=Gestational age, CS=cesarean sectio, PC=Principal Component. Categorical values of CS and emergency CS where a case of CS and emergency CS respectively are the references.

**^1^** model was adjusted for maternal BMI and protein level at baseline and w10-14, PC1-PC3 (omitted in the Table).

**Table S6. Associations between protein change from baseline to w 26-28 and birth outcomes**

|  | **PC1^1^** | | **PC2^1^** | | **PC3^1^** | | **PC1^1^** | | **PC2^1^** | | **PC3^1^** | |
| --- | --- | --- | --- | --- | --- | --- | --- | --- | --- | --- | --- | --- |
| **Outcome** | ***β* ∆** | **p** | ***β* ∆** | **p** | ***β* ∆** | **p** | **OR ∆** | **p** | **OR ∆** | **p** | **OR ∆** | **p** |
| **BW** | 33.83 | 0.26 | -14.79 | 0.71 | -10.09 | 0.81 |  |  |  |  |  |  |
| **GA** | 0.02 | 0.98 | 0.32 | 0.76 | -0.44 | 0.70 |  |  |  |  |  |  |
| **CS** |  |  |  |  |  |  | 1.14 | 0.39 | 0.81 | 0.33 | 0.80 | 0.31 |
| **Emergency CS** |  |  |  |  |  |  | 1.06 | 0.78 | 0.79 | 0.40 | 0.75 | 0.29 |

*β*=Beta-coefficient, ∆= difference between baseline and w26-28, OR=Odds Ratio, BW=Birthweight, GA=Gestational age, CS=cesarean sectio, PC=Principal Component. Categorical values of CS and emergency CS where a case of CS and emergency CS respectively are the references. Protein change is the difference between PC at baseline and PC at w 26-28.

**^1^** model was adjusted for maternal BMI (omitted in the Table).
